# Protective Effect of Insulin in Mouse Nasal Mucus Against Olfactory Epithelium Injury

**DOI:** 10.3389/fncir.2021.803769

**Published:** 2021-12-23

**Authors:** Shu Kikuta, Akihito Kuboki, Tatsuya Yamasoba

**Affiliations:** ^1^Department of Otorhinolaryngology and Head and Neck Surgery, Graduate School of Medicine, The University of Tokyo, Bunkyo, Japan; ^2^Department of Otorhinolaryngology, The Jikei University School of Medicine, Minato, Japan

**Keywords:** odor, olfactory dysfunction, olfactory sensory neuron, insulin, chronic rhinosinusitis (CRS)

## Abstract

Insulin is present in nasal mucus and plays an important role in the survival and activity of individual olfactory sensory neurons (OSNs) *via* insulin receptor-mediated signaling. However, it is unclear whether insulin acts prophylactically against olfactotoxic drug-induced olfactory epithelium (OE) injury, and whether the degree of damage is affected by the concentration of insulin in the nasal mucus. The apoptosis-inducing drug methimazole was administered to the nasal mucus of diabetic and normal mice along with different concentrations of insulin. Immunohistochemical analysis was used to assess the relationship between damage to the OE and the mucus insulin concentration and the protective effect of insulin administration against eosinophilic cationic protein (ECP)-induced OE injury. Diabetic mice had lower concentrations of insulin in their nasal mucus than normal mice (diabetic vs. normal mice, *p* < 0.001). Methimazole administration reduced the number of OSNs in normal mice and had a more marked effect in diabetic mice. However, unilateral insulin administration prevented the methimazole-induced reduction in the number of OSNs on the ipsilateral side but not on the contralateral side (OSNs; Insulin vs. contralateral side, *p* < 0.001). Furthermore, intranasal ECP administration damaged the OE by inducing apoptosis (OSNs; ECP vs. contralateral side, *p* < 0.001), but this damage was largely prevented by insulin administration (OSNs; Insulin + ECP vs. contralateral side, *p* = 0.36), which maintained the number of mature OSNs. The severity of methimazole-induced damage to the OE is related to the insulin concentration in the nasal mucus (Correlation between the insulin concentration in nasal mucus and the numbers of OSNs, *R*^2^ = 0.91, *p* < 0.001), which may imply that nasal insulin protects OSNs and that insulin administration might lead to the development of new therapeutic agents for ECP-induced OE injury.

## Introduction

Olfactory sensory neurons (OSNs) are essential for odor sensing, which helps maintain a healthy lifestyle by preventing the ingestion of tainted foods and entry into dangerous environments while allowing the recognition of edible food and familiar scents. However, respiration-mediated repeated exposures of OSNs to environmental chemicals and viral and bacterial pathogens often damages OSNs at an estimated incidence of between 3 and 20% of the general population (Boesveldt et al., 2017).

As a central homeostatic mechanism of odor sensing, OSNs are continuously replaced with newly generated OSNs (Litvack et al., 2009; Soler et al., 2010; Whitcroft et al., 2018; Cooper et al., 2020). Despite OSN turnover, chronic rhinosinusitis (CRS) or some infectious diseases sometimes reduce or result in loss of smell in patients *via* mucosal inflammation (Ahmed and Rowan, 2020; Cooper et al., 2020). In fact, CRS is the most frequent etiology of olfactory dysfunction (Kohli et al., 2017). In particular, CRS with polyps, which is usually characterized by mucosal type 2 inflammation, is associated with a high prevalence of olfactory dysfunction (Yan et al., 2020). The accompanying eosinophilia is associated with the release of several neurotoxins, such as eosinophilic cationic protein (ECP), major basic protein, and β-glucuronidase. These substances can induce the apoptosis of OSNs, and their concentrations are quantitatively associated with olfactory dysfunction (Becker et al., 2012). In addition, when the OE is severely damaged or persistently exposed to neurotoxic substances, the olfactory impairment becomes chronic, despite the unique neural plasticity of the OE (Litvack et al., 2009; Soler et al., 2010; Whitcroft et al., 2018; Cooper et al., 2020). Therefore, it is important to protect OSNs from toxic compounds and prevent pathological conditions in order to maintain olfactory function.

Insulin is a peptide hormone produced by pancreatic beta cells that principally regulates metabolism in the periphery. However, insulin and insulin receptors are also present in the central nervous system. Insulin influences brain development by affecting the proliferation, survival, and differentiation of neurons, and by modulating neuronal circuits in the brain (Fernandez and Torres-Alemán, 2012).

Insulin is also present in nasal mucus and influences the maturation and activity of individual OSNs through insulin receptor-mediated signaling (Henkin, 2010; Kuboki et al., 2021). Further, the apoptotic wave that occurs after interruption of the neuronal connections between the OE and the olfactory bulb following bulbectomy is impaired by intranasal insulin administration (Lacroix et al., 2011). However, it is unclear whether insulin acts prophylactically against different types of injury to the OE, and whether the degree of injury is affected by the concentration of insulin in the nasal mucus.

In this study, we examined the effects of insulin on two types of OSN damage, methimazole and neurotoxic ECP-induced apoptosis in normal and diabetic mice. The results showed that methimazole-induced OE damage was inhibited by insulin administration; a higher insulin concentration resulted in a higher number of remaining OSNs, while a lower concentration resulted in a lower number of remaining OSNs. Furthermore, ECP-induced OE injury was largely inhibited by insulin administration. These results indicate that the degree of OE damage strongly depended on the concentration of insulin in the nasal mucus before injury.

## Materials and Methods

### Animals

We studied male, 10-week-old C57BL/6 mice, which were exposed to a light-dark cycle (lights on, 6 a.m.; lights off, 6 p.m.). Mice were randomly assigned to each experimental group.

The study was performed using procedures approved by the Experimental Animal Research Committee of the University of Tokyo. All experiments were carried out in compliance with the ARRIVE guidelines.

The pancreatic beta cells of mice were ablated by intraperitoneal (i.p.) injections of streptozotocin (STZ) (120 mg/kg; Sigma Aldrich, St. Louis, MO, United States) dissolved in 0.9% NaCl solution (saline) on 3 consecutive days. Seven days after the first STZ injection, the fasting tail blood glucose concentrations of the mice were measured using a glucose reader (Ascensia Diabetes Care, Basel, Switzerland). Mice were considered to be diabetic if their fasting blood glucose concentrations were ≥ 250 mg/dl.

The OSNs of normal and STZ-treated diabetic mice were ablated by the i.p. injection of methimazole (35 mg/kg; Sigma-Aldrich) dissolved in saline. The mice were perfused with fixative 4 days after the methimazole administration. Methimazole disrupts the OE in a volume-dependent manner (Kikuta et al., 2015). The dose used in this study was about half that used previously (Kuboki et al., 2021).

Pilocarpine (0.1 mg/kg; Fujifilm Wako Pure Chemical Corporation, Osaka, Japan) was intraperitoneally injected to increase mucus secretion at a fixed time before 6 p.m., and samples of nasal secretions were collected 20 min after injection (Kikuta et al., 2016).

### Intranasal Insulin Administration

**FIGURE 1:**
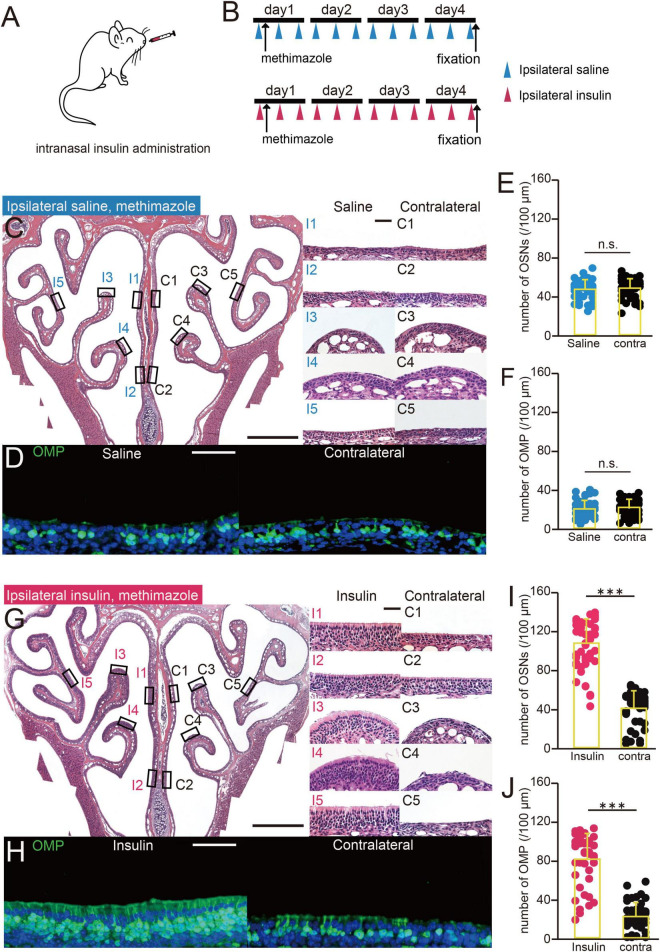
Increasing the concentration of insulin in nasal mucus reduces damage to the OE. **(A)** Diagram of intranasal insulin administration. Insulin was administered to one nostril after methimazole-induced injury. **(B)** Experimental design. The mice were administered saline or insulin intranasally unilaterally three times a day after methimazole-induced injury. Both groups were administered methimazole intraperitoneally on day 1, and intranasal saline (upper group) or insulin (lower group) from day 1. The mice were perfused with fixative on day 4. **(C)** Photomicrographs of representative coronal sections 4 days following the administration of methimazole to mice administered saline unilaterally. Left, lower magnification; right, higher magnification of that part of the OE (I, saline-administered side; C, contralateral side; 1, upper nasal septum; 2, lower nasal septum; 3, upper turbinate; 4, lower turbinate; 5, lateral turbinate). Scale bars, 50 μm at higher magnification, 500 μm at lower magnification. **(D)** Photomicrographs of representative coronal sections at day 4 following the administration of methimazole in unilaterally saline-administered mice, immunostained for OMP. Green, OMP; blue, DAPI. Scale bar, 50 μm. **(E,F)** Numbers of OSNs **(E)** and OMP-positive cells **(F)** at day 4 following the administration of methimazole to unilaterally saline-treated mice. There were no significant differences in the numbers of OSNs or OMP-positive cells between the treated (blue) and contralateral (black) sides (OSNs, *p* = 0.43; OMP, *p* = 0.32, Mann–Whitney *U-*test). n.s., no significant difference. **(G)** Photomicrographs of representative coronal sections at day 4 following the administration of methimazole to unilaterally insulin-treated mice. Left, lower magnification; right, higher magnification of that part of the OE (I, insulin-administered side; C, contralateral side). Scale bars, 50 μm at higher magnification, 500 μm at lower magnification. **(H)** Photomicrographs of representative coronal sections 4 days following the administration of methimazole to unilaterally insulin-treated mice, immunostained for OMP. Green, anti-OMP; blue, DAPI. Scale bar, 50 μm. **(I,J)** Numbers of OSNs **(I)** and OMP-positive cells **(J)** 4 days following the administration of methimazole to unilaterally insulin-administered mice. There were significant differences in the numbers of OSNs and OMP-positive cells between the treated (red) and contralateral (black) sides (Mann–Whitney *U-*test, ^***^ *p* < 0.001).

Saline was administered to one group of hand-restrained, supine mice as one 30 μl drop into a single nostril (Figure 1C), and insulin diluted 1:3 in saline was administered unilaterally to another group of mice as one 30 μl drop (Humulin R, 100 units/ml; Eli Lilly, Indianapolis, IN, United States) into a single nostril (Figure 1G). The fluid was taken into the nasal passage by the animal’s natural inhalation. This process was repeated three times daily, resulting in the delivery of a total of 90 μl insulin solution or saline, as previously described (Kuboki et al., 2021).

### Intranasal Saline/Insulin + Eosinophilic Cationic Protein Administration

**FIGURE 2:**
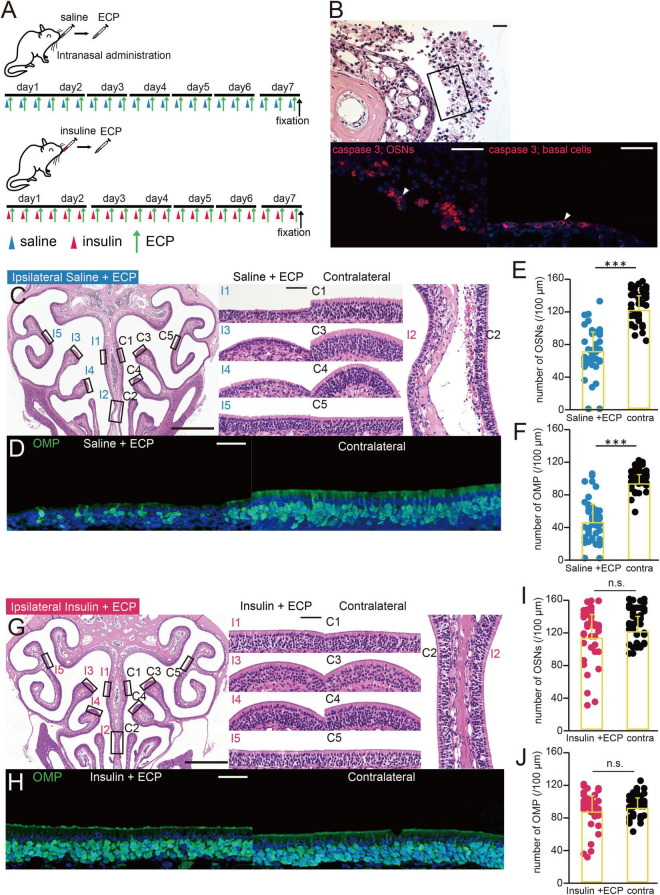
ECP-induced apoptosis is inhibited by insulin administration. **(A)** Experimental design. The mice were administered saline or insulin in one nostril prior to the intranasal administration of eosinophilic cationic protein (ECP). A combination of saline/ECP or insulin/ECP was administered for 7 consecutive days, after which the mice were perfused with fixative. **(B)** Photomicrographs of representative coronal sections after 7 days of administration of ECP. Upper, lower magnification of olfactory sensory neurons (OSNs); lower left, higher magnification of that part of the OSNs, immunostained for caspase 3; lower right, basal progenitor cells, immunostained for caspase 3. Arrowheads indicate caspase 3-positive cells. Scale bar, 50 μm. **(C)** Photomicrographs of representative coronal sections after 7 days of administration of saline and ECP to mice. Left, lower magnification of the OE; right, higher magnification of that part of the OE (I, saline + ECP-administered side; C, contralateral side; 1, upper nasal septum; 2, lower nasal septum; 3, upper turbinate; 4, lower turbinate; 5, lateral turbinate). Scale bars, 50 μm at higher magnification, 500 μm at lower magnification. **(D)** Photomicrographs of representative coronal sections after 7 days of administration of saline and ECP to mice, immunostained for OMP. Green, OMP; blue, DAPI. Scale bar, 50 μm. **(E,F)** Numbers of OSNs **(E)** and OMP-positive cells **(F)** after 7 days of administration of saline and ECP to mice. ECP administration significantly reduced the numbers of OSNs and OMP-positive cells. Treated side, blue; contralateral side, black. ^***^p < 0.001, Mann–Whitney *U*-test. **(G)** Photomicrographs of representative coronal sections after 7 days of administration of insulin and ECP to mice. Left, lower magnification; right, higher magnification of that part of the OE (I, insulin + ECP-administered side; C, contralateral side). Scale bars, 50 μm at higher magnification, 500 μm at lower magnification. **(H)** Photomicrographs of representative coronal sections after 7 days of administration of insulin and ECP to mice, immunostained for OMP. Green, OMP; blue, DAPI. Scale bar, 50 μm. **(I,J)** Numbers of OSNs **(I)** and OMP-positive cells **(J)** after 7 days of administration of insulin and ECP to mice. Insulin administration prior to ECP administration did not affect the numbers of OSNs and OMP-positive cells. Treated side, red; contralateral side, black. OSNs, *p* = 0.36; OMP, *p* = 0.45, Mann–Whitney *U-*test. n.s., no significant difference.

One 30 μl drop of saline alone (Figure 2C) or insulin (Humulin R, 100 units/ml, Figure 2G) diluted 1:3 in saline was administered into a single nostril, while the animal was hand-restrained in a supine position. Three hours later, one 30 μl drop of ECP (3 μg/ml in saline, Mouse Eosinophilic Cationic Protein Recombinant; Aviscera Bioscience, Santa Clara, CA, United States) was administered into the same nostril used for saline or insulin administration. This process was repeated three times daily. The mice were perfused with fixative 7 days after the administration of ECP commenced.

### Enzyme-Linked Immunosorbent Assay

Mucus insulin concentration was determined using a Mouse Ultrasensitive Insulin ELISA kit (Mercodia, Uppsala, Sweden), according to the manufacturer’s instructions. Mucus samples were collected at the same time each day.

### Immunohistochemistry

The mice were perfused intracardially with 4% paraformaldehyde in 0.1 M phosphate buffer, decapitated, and then post-fixed for 24 h in the same fixative. Their nasal tissues, including the OE, were decalcified using 10% EDTA solution, pH 7.0, and then embedded in paraffin. Coronal sections (4 μm thick) were prepared and mounted on silane-coated slides, deparaffinized, and then autoclaved for 10 min in Target Retrieval Solution (S1700; Dako, Santa Clara, CA, United States) for antigen retrieval (Kikuta et al., 2015; Kuboki et al., 2021).

**FIGURE 3:**
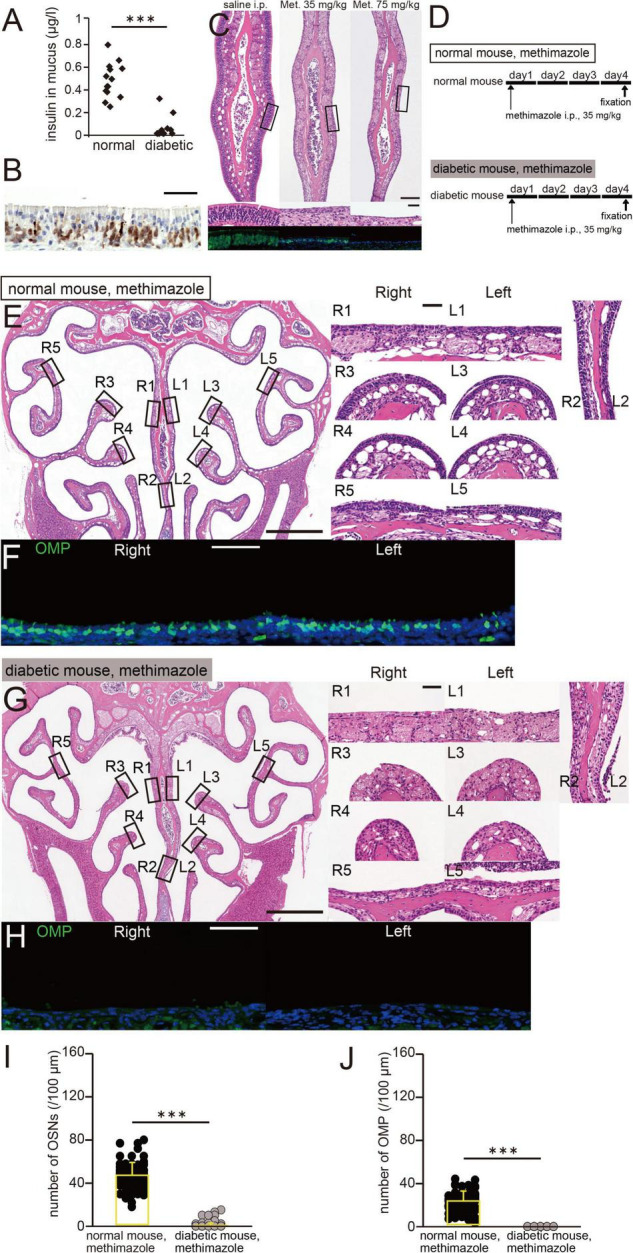
A low insulin concentration in nasal mucus is associated with more OE damage. **(A)** Insulin concentration in the nasal mucus of normal and diabetic mice. Pilocarpine was intraperitoneally administered to induce nasal mucus secretion. Normal mice, *n* = 14; diabetic mice, *n* = 12 mice. ^***^p < 0.001, Mann–Whitney *U-*test. **(B)** Photomicrograph of a representative coronal section of the olfactory epithelium (OE) 6 h after the administration of methimazole to normal mice (75 mg/kg). The coronal section was stained with hematoxylin and anti-caspase-3 antibody. Scale bar, 50 μm. **(C)** Photomicrographs of representative coronal sections of the OE, stained with hematoxylin (upper, lower magnification; middle, higher magnification of the same part of the OE) and anti-OMP antibody (lower, green), in saline-treated (left) and methimazole-treated mice (middle, 35 mg/kg; right, 75 mg/kg). Scale bar, 50 μm. **(D)** Time course and experimental design. Methimazole administration was performed on day 1, and perfusion with fixative was performed on day 4, in methimazole-treated normal and diabetic mice. **(E)** Photomicrographs of representative coronal sections 4 days following the administration of methimazole to normal mice. Left, lower magnification; right, higher magnification of the same part of the OE (R, right side; L, left side; 1, upper nasal septum; 2, lower nasal septum; 3, upper turbinate; 4, lower turbinate; and 5, lateral turbinate). Scale bars, 50 μm at higher magnification, 500 μm at lower magnification. **(F)** Photomicrographs of representative coronal sections after 4 days of administration of methimazole to normal mice, immunostained for olfactory marker protein (OMP). Green, anti-OMP; blue, DAPI. Scale bar, 50 μm. **(G)** Photomicrographs of representative coronal sections after 4 days of administration of methimazole to diabetic mice. Left, lower magnification; right, higher magnification of the same part of the OE. Scale bars, 50 μm at higher magnification, 500 μm at lower magnification. **(H)** Photomicrographs of representative coronal sections after 4 days of administration of methimazole to diabetic mice, immunostained for OMP. Green, OMP; blue, DAPI. Scale bar, 50 μm. **(I,J)** Numbers of OSNs **(H)** and OMP-positive cells **(I)** after 4 days of administration of methimazole to normal (black) and diabetic (gray) mice. There were significantly fewer OSNs and mature OSNs in diabetic mice than in normal mice (Mann–Whitney *U-*test, ^***^p < 0.001).

Immunohistochemistry was performed using one of the following antibodies: anti-olfactory marker protein (OMP; goat polyclonal, 1:4,000 dilution; Fujifilm Wako Pure Chemical Corporation, Osaka, Japan) or anti-activated caspase-3 (rabbit polyclonal, 1:500; Cell Signaling Technology, Danvers, MA, United States). After overnight incubation, the sections were washed with PBS and incubated with the following secondary antibodies for 1 h at room temperature, as described previously (Kuboki et al., 2021): donkey anti-goat Alexa Fluor 488 (1:100; Invitrogen, Waltham, MA, United States; A32814, RRID: AB_2762838) and donkey anti-rabbit Alexa Fluor 594 (1:100; Invitrogen, A21207, RRID: AB_141637). Nuclei were stained using 4′,6-diamidino-2-phenylindole (DAPI; 0.1 μg/ml, D3571; Life Technologies, Carlsbad, CA, United States). The immunoreaction (Figure 3B) was detected using the Histofine Simple Stain MAX-PO secondary antibody system (Nichirei Corp., Tokyo, Japan) for anti-activated caspase-3 (rat) and the CSA II kit from Dako Japan Inc. (Tokyo, Japan), a biotin-free tyramide signal amplification system, according to the manufacturers’ instructions. Images were acquired using a fluorescence microscope (BZ*-*X710; Keyence, Osaka, Japan*).*

### Analysis

**FIGURE 4:**
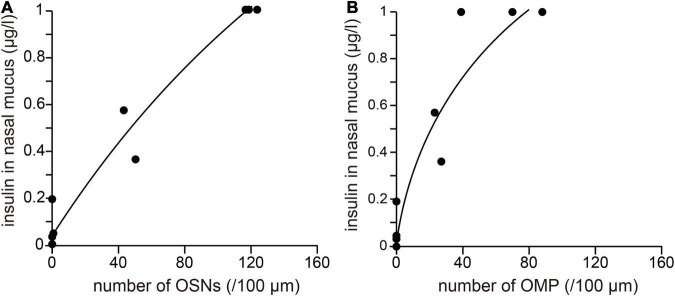
The degree of OE damage is related to the concentration of insulin in the nasal mucus. **(A,B)** Correlation between the insulin concentration in nasal mucus and the numbers of OSNs **(A)** and OMP-positive cells **(B)**. Number of OSNs: *R*^2^ = 0.91, ^***^ *p* < 0.001; number of OMPs: *R*^2^ = 0.92, ^***^ *p* < 0.001.

The OE contains three major cell types: OSNs, supporting cells, and basal progenitor cells. The supporting cells were defined as the apical columnar cells, the basal progenitor cells as the rectangular cells on the basal lamina. The remaining cells were defined as OSNs. The numbers of OSNs and immunostained cells (OMP-positive cells) were counted on three coronal sections, spaced 500 μm apart, between the caudal and rostral regions of the OE. The numbers of OSNs and immunostained cells (OMP-positive cells) were counted in three coronal sections, spaced 500 mm apart, between the caudal and rostral regions of the OE, from at least three mice (Kikuta et al., 2015). Immunostaining of cells that exceeded two standard deviations (SDs) from the mean background intensity for the connective tissue deep to the basal lamina was considered positive. To analyze a broader area of the OE, coronal sections were divided into ten regions (five per side): 1, upper nasal septum; 2, lower nasal septum; 3, upper turbinate; 4, lower turbinate; and 5, lateral turbinate. The numbers of OSNs and OMP-positive cells per 100 μm length of the OE in each region were counted, and the mean ± SD number of counts was then calculated for each experimental group. To evaluate the relationship between insulin concentration and the degree of OE damage (Figure 4), we performed correlation analysis using the mean number of OSNs or OMP-positive OSNs in the treated nostril of each mouse. The experimenter who performed the analyses was blinded to the identity of each specimen.

### Statistical Analysis

The data were statistically analyzed using OriginPro software (OriginLab Corporation, Northampton, MA, United States) and JMP Statistical Discovery software (SAS Institute Japan, Tokyo, Japan). Statistical analyses were performed using Mann–Whitney *U-*tests (Figures 1E,F,I,J, 2E,F,I,J, 3A,I,J). Spearman’s rank correlation coefficients were calculated for the data shown in Figure 4. All values are expressed as mean ± SD. *P* < 0.05 was considered to represent statistical significance.

## Results

### A Low Insulin Concentration in the Nasal Mucus Is Associated With More Olfactory Epithelium Damage

STZ was intraperitoneally injected to selectively destroy pancreatic beta cells, as previously described (Kuboki et al., 2021). The mucus insulin concentration of diabetic mice was significantly lower than that of normal mice (normal, *n* = 14 mice; diabetic, *n* = 12 mice, *p* < 0.001, Mann–Whitney *U-*test; Figure 3A). The olfactotoxic drug, methimazole disrupts virtually all OSNs across the OE by activating an apoptotic cascade (Sakamoto et al., 2007; Kikuta et al., 2015). A representative picture of the OE 6 h after methimazole administration (75 mg/kg) is shown in Figure 3B. Consistent with previous report (Sakamoto et al., 2007), we observed that a large number of OSNs underwent apoptotic cell death upon methimazole administration. In normal untreated mice, OSNs were densely packed in multiple rows above the basal progenitor cells (Figure 3C, left). However, when methimazole at a concentration of 35 mg/kg was administered, OSNs underwent apoptosis, such that the thickness of the OE after 3 days of administration was approximately half that of controls, and the number of OSNs was much lower (Figure 3C, middle). Alongside the lower number of OSNs, the number of cells that were positive for OMP, a marker of mature OSNs, was also lower. When methimazole at a concentration of 75 mg/kg was administered, most OSNs underwent substantial apoptosis, but the basal progenitor cells remained intact (Figure 3C, right). OSNs that were positive for OMP could no longer be observed through the OE. These results indicate that the degree of damage to the OE increased with the concentration of methimazole administered.

We next determined whether a low insulin concentration in nasal mucus would be associated with greater susceptibility to methimazole-induced OE injury. The experimental protocol is shown in Figure 3D (upper, methimazole administration to normal mice; lower, methimazole administration to diabetic mice). Methimazole administration at a concentration of 35 mg/kg to normal mice also reduced the number of OSNs in each region (R, right side; L, left side; 1, upper nasal septum; 2, lower nasal septum; 3, upper turbinate; 4, lower turbinate; and 5, lateral turbinate; Figure 3E). The number of OMP-positive cells was also lower in the nasal passages of both groups of mice (Figure 3F). OE thickness and the number of OSNs did not differ between diabetic mice and control mice (Kuboki et al., 2021), suggesting that decreased insulin signaling alone does not induce histological changes in the OE. However, in methimazole-treated diabetic mice, there were far fewer OSNs in each region of the OE (Figure 3G), and no mature OSNs were visible (Figure 3H). In detail, the numbers of OSNs and OMP-positive cells in the methimazole-treated diabetic mice were significantly lower than in the methimazole-treated normal mice (normal mice, *n* = 3; diabetic mice, *n* = 4 mice, OSNs, *p* < 0.001, Figure 3I; OMP, *p* < 0.001, Figure 3J, Mann–Whitney *U-*test). These results suggest that a low concentration of insulin in the nasal mucus is associated with greater susceptibility to methimazole-induced OE injury.

### Increasing the Concentration of Insulin in Nasal Mucus Reduces Damage to the Olfactory Epithelium

We next determined whether increasing the nasal concentration of insulin would reduce susceptibility to methimazole-induced OE injury (Figure 1A). Saline or insulin was administered to the same nostril three times a day for 4 days, in parallel with methimazole administration at a concentration of 35 mg/kg on day 1, as shown in Figure 1B. In mice administered saline for 4 days, the OE was thinner in all its regions (Figure 1C), and OMP-positive cells were rare in the bilateral OE (Figure 1D). There were no significant differences in the numbers of OSNs or mature OSNs between the nasal passages in saline-treated mice (*n* = 3 mice, OSNs, *p* = 0.43, Figure 1E; OMP, *p* = 0.32, Figure 1F, Mann–Whitney *U-*test). However, in mice treated with insulin for 4 days, the OE structure was largely unimpaired by methimazole-induced injury, as evidenced by the presence of numerous OSNs on a wide area of the OE and the absence of an effect on the thickness of the OE on the insulin-treated side (Figure 1G). OMP-positive cells were also numerous in the OE (Figure 1H). The analysis showed that the numbers of OSNs and OMP-positive cells were significantly higher on the insulin-treated side than on the contralateral side (*n* = 3 mice, OSNs, *p* < 0.001, Figure 1I; OMP, *p* < 0.001, Figure 1J, Mann–Whitney *U-*test). These results indicate that increasing the intranasal concentration of insulin reduces methimazole-induced OE injury.

### The Degree of Olfactory Epithelium Damage Is Related to the Concentration of Insulin in the Nasal Mucus

The relationships between the concentration of insulin in nasal mucus and the numbers of OSNs or mature OSNs present after methimazole administration are shown in Figure 4. The *x*-axis indicates the mean numbers of OSNs or mature OSNs after methimazole administration into one nasal passage (methimazole-treated normal mice, *n* = 3; methimazole-treated diabetic mice, *n* = 2; methimazole + unilateral nasal insulin administration to normal mice, *n* = 2), and the *y*-axis indicates the concentration of insulin in the nasal mucus. There were strong positive correlations between the insulin concentration and the numbers of OSNs (*R*^2^ = 0.96, *p* < 0.001; correlation coefficient; Figure 4A) or mature OSNs (*R*^2^ = 0.91, *p* < 0.001; correlation coefficient; Figure 4B), which implies that susceptibility to methimazole-induced OE injury is related to the nasal concentration of insulin.

### Eosinophilic Cationic Protein-Induced Apoptosis Is Inhibited by Insulin Administration

A mouse model of olfactory dysfunction due to CRS with polyps was created by ECP administration. To determine whether damage to the OE caused by ECP administration decreased after insulin administration, saline or insulin was unilaterally administered in parallel with ECP, which was ipsilaterally administered three times a day for 7 days, as shown in Figure 2A. After ECP administration for 7 days, many of the OSNs had detached from the basal lamina and were caspase 3-positive, as in methimazole-treated mice (Figure 2B). Some basal progenitor cells were also observed to be positive for caspase-3 (Figure 2B). However, no polyp formation or polyp-like changes were observed in the OE.

In mice that had been administered saline alongside ECP, the OE was thinner on the treated side (Figure 2C) and there were fewer OMP-positive cells (Figure 2D). In addition, the numbers of OSNs and mature OSNs on the treated side were significantly lower than on the contralateral side (*n* = 3 mice, OSNs, *p* < 0.001, Figure 2E; OMP, *p* < 0.001, Figure 2F, Mann–Whitney *U*-test). By contrast, in mice that had been unilaterally administered insulin alongside ECP, the OE on the treated side remained thicker (Figure 2G) and there were numerous mature OSNs (Figure 2H). Indeed, there were no differences in the numbers of OSNs and mature OSNs between the nasal passages (*n* = 3 mice, OSNs, *p* = 0.36, Figure 2I; OMP, *p* = 0.45, Figure 3J, Mann–Whitney *U*-test). These results suggest that increasing the nasal concentration of insulin prevents the induction of apoptosis by ECP, which contributes to the protective effect of nasal insulin against ECP-induced olfactory disorders.

## Discussion

We previously reported that, after injury, insulin receptor signaling plays an important role in the maturation of newly generated OSNs when insulin receptor signaling and insulin receptor expression are highly upregulated. Furthermore, nasal insulin application accelerates OE regeneration (Kuboki et al., 2021). In contrast to the previous report, in this study, we investigated whether insulin prevents the occurrence of OE injury. We found that the degree of methimazole-induced damage to the total OE including immature OSNs depended on the insulin concentration in the nasal mucus. Furthermore, ECP-induced cell death was largely prevented by insulin administration. Thus, insulin receptors might actually be constitutively activated in the uninjured OE, and insulin might have a novel function in the nasal mucus in protecting OSNs against various toxic insults.

In addition to methimazole administration, bulbectomy is also known as a model of the OE damage. Both methods commonly induce apoptotic cell death triggered primarily through mitochondrial cytochrome *c*-mediated caspase-3 activation pathway, but the timing of apoptosis is different. After bulbectomy, the proapoptotic signals by caspase-3 were propagated from the axonal end to the cell body of the OSNs, resulting that activation of caspase-3 is highest after 48 h after treatment (Holcomb et al., 1995; Cowan et al., 2001). By contrast, in methimazole-induced apoptosis, methimazole is taken up into the Bowman’s glands and the supporting cells within 30 min after injection (Bergman and Brittebo, 1999). Therefore no delay due to propagation occurs and activation of caspase-3 is observed within 24 h after treatment (Sakamoto et al., 2007). The detailed mechanism of ECP-induced damage and the timing of cell death after ECP administration need to be investigated.

When insulin is administered intranasally at high concentration, it can spillover into the peripheral blood, which can result in an increase in insulin concentration in peripheral blood and a concomitant decrease in blood glucose levels (Kuboki et al., 2021). Because fluctuations in blood glucose levels may affect susceptibility to OE injury (Giacco and Brownlee, 2010), we determined the insulin concentration that changes the insulin concentration in the nasal mucus without changing the blood glucose level in a previous study (Kuboki et al., 2021) and used it in the present study.

Insulin is produced in the developing rodent brain, but whether this hormone is produced in the adult brain remains controversial (Banks, 2004). Although one report suggested that insulin might be produced by the OE (Lacroix et al., 2008), the current consensus is that no insulin is produced by adult mammalian neurons (Schwartz et al., 2010; Fernandez and Torres-Alemán, 2012). Further, in STZ-induced diabetic mice, insulin levels in the nasal mucus were extremely low (Figure 3). Accordingly, we think that the primary source of insulin in the nasal mucus is pancreatic beta cells.

A series of defensive mechanisms have evolved to protect airways from insults (Dickson and Huffnagle, 2015). The surface of the OE is covered with mucus, which traps inhaled particles and foreign pathogens, lubricates airway surfaces, and facilitates the function of the cilia that clear the mucus efficiently (Wanner et al., 1996). At the cellular level, OSNs contains various antioxidants and chemo-protective enzymes, such as NADPH quinone oxido-reductase, glutathione peroxidase, and superoxide dismutase (Imamura and Hasegawa-Ishii, 2016). However, in addition, insulin in nasal mucus might help to protect the OE, and increasing the mucus insulin concentration might reduce the susceptibility of the OE to damage.

Beneficial effects of intranasal insulin administration have been shown in clinical trials of its use to improve memory and cognition, and to reduce the memory loss caused by Alzheimer’s disease and diabetes (Kern et al., 1999; Benedict et al., 2008; Reger et al., 2008). Furthermore, intranasal insulin administration has been shown to neither have harmful side effects nor cause a change in blood glucose concentration (González et al., 2006). However, despite this evidence of clinical efficacy and safety, the potential protective effect of insulin on OSNs has not been investigated.

In the present study, we used a mouse model of ECP-induced damage to the OE to determine whether intranasal insulin could prevent a CRS-induced olfactory disorder. This was characterized by substantial apoptosis of OSNs and apoptosis of some basal progenitor cells. Once the basal progenitor cells are damaged, the OE can no longer regenerate, which leads to irreversible olfactory dysfunction and may explain why patients with CRS and polyps have a poor olfactory prognosis following endoscopic sinus surgery (Haxel, 2019). In addition to neurotoxic mediators released from eosinophils, the loss of smell in CRS may be caused by mucosal type 2 inflammation. Local overexpression of pro-inflammatory cytokines, such as tumor necrosis factor alpha (TNF-α), interferon gamma (IFN-γ), and certain cytokines, including interleukin (IL)-2, IL-5, IL-6, IL-10, and IL-13, also adversely affects the OE (Turner et al., 2010; Yee et al., 2010; Pozharskaya and Lane, 2013; Haxel, 2019). Importantly, regardless of the type of neurotoxic mediator or inflammatory cytokine present, OSNs undergo inflammation-induced apoptotic cell death (Islam et al., 2007; Lane et al., 2010; Turner et al., 2010; Yee et al., 2010; Pozharskaya and Lane, 2013; Kanaya et al., 2014; Sousa Garcia et al., 2017). Therefore, limiting the apoptosis of OSNs represents a key therapeutic strategy for olfactory disorders.

Intracellular signaling *via* the insulin receptor involves the phosphoinositide 3-kinase (PI3K)-AKT-forkhead box protein O and RAS-mitogen-activated protein kinase pathways, which regulate gene transcription and activate myriad downstream kinases/phosphatases that ultimately affect a range of key cellular processes, including autophagy, apoptosis, and resistance to oxidative stress (Fernandez and Torres-Alemán, 2012). Therefore, increasing the concentration of insulin in nasal mucus may reduce activation of the intrinsic apoptosis activation pathway *via* the insulin receptor on OSNs, and the suppression of OSN apoptosis may be a key mechanism whereby insulin protects the OE from injury.

A loss of caspase activation because of an increase in neural activity may also be involved in olfactory neuroprotection. Neuronal activity normally involves the generation of action potentials, which is mediated by an increase in cytoplasmic cAMP concentration (Lessmann et al., 2003; Park and Poo, 2013). cAMP also activates intracellular PI3K/AKT signaling and triggers the production of extracellular signals, such as neurotrophins, cytokines, and glial-derived neurotropic factor, which inhibit the intrinsic apoptosis machinery (Raff, 1992; Frade et al., 1996; Nunez and del Peso, 1998; Watt et al., 2004; Hyman and Yuan, 2012). Insulin markedly increases the spontaneous generation of action potentials, as shown in rat patch-clamp experiments (Savigner et al., 2009), which increases the activity of OSNs and may lead to greater production of anti-apoptotic signals that act on OSNs. If intranasal insulin administration could reduce the apoptosis of OSNs through insulin receptor-mediated signaling and/or activity-mediated cAMP-related signaling, it might prevent OE damage induced by various neurotoxic factors, such as viruses and harmful chemicals. Furthermore, because olfactory dysfunction in CRS with polyps is caused by continuous exposure to neurotoxins or pro-inflammatory cytokines, insulin administration may prevent excessive apoptosis of OSNs in the OE, facilitating a restoration of the sense of smell, secondary to OSN regeneration.

With regard to the site of insulin action, the hormone may target neurons in the olfactory bulb (OB), in addition to OSNs in the OE. Chronic nasal inflammation including CRS causes glial activation and a reduction in thickness of the superficial layers of the OB, owing to the loss of OSN axons and the apoptosis of juxtaglomerular cells (Rombaux et al., 2008; Hasegawa-Ishii et al., 2019). Furthermore, a number of viruses can pass from the OE through the cribriform plate to infect the OB and induce neuronal death (Potter et al., 2020). This implies that part of the central nervous system may also be affected in chronic nasal inflammation and post-infectious olfactory disorders. Insulin is extracellularly transferred by OSN axons *via* the lamina cribrosa to the OB (Crowe et al., 2018), which is rich in insulin receptors (Fernandez and Torres-Alemán, 2012). Thus, intranasal insulin may support the formation of stable neural circuits by inhibiting the apoptosis of OB neurons, as well as by protecting the OE.

Future studies will be required to obtain a better understanding of the molecular and cellular mechanisms involved in the regulation of OE homeostasis by neurotrophic factors and insulin receptor signaling, as well as to elucidate the pharmacokinetics of insulin in the nasal mucus, to assess the potential for the development of novel therapeutic agents for CRS-related olfactory dysfunction.

## Conclusion

In the present study, we determined whether insulin in nasal mucus protects against injury to the OE and explored whether it might represent a new therapeutic agent for CRS-related olfactory dysfunction. The degree of OE damage induced depended on the concentration of insulin in the nasal mucus of mice: Higher insulin concentrations were associated with the preservation of more OSNs, and vice versa. Furthermore, ECP-induced OE damage was inhibited by insulin administration. These results suggest that mucus insulin has a protective effect on OSNs, and that insulin administration might represent a means of preventing ECP-induced OE damage.

## Data Availability Statement

The raw data supporting the conclusions of this article will be made available by the authors, without undue reservation.

## Ethics Statement

The animal study was reviewed and approved by the Experimental Animal Research Committee at the University of Tokyo.

## Author Contributions

SK and AK designed the studies. SK performed the experiments, analyzed the data, created the drawings in the figures, and wrote the manuscript. SK, AK, and TY gave technical support and conceptual advice, revised, and finalized the manuscript. All authors read and approved the manuscript.

## Conflict of Interest

The authors declare that the research was conducted in the absence of any commercial or financial relationships that could be construed as a potential conflict of interest.

## Publisher’s Note

All claims expressed in this article are solely those of the authors and do not necessarily represent those of their affiliated organizations, or those of the publisher, the editors and the reviewers. Any product that may be evaluated in this article, or claim that may be made by its manufacturer, is not guaranteed or endorsed by the publisher.
